# AflaILVB/G/I and AflaILVD are involved in mycelial production, aflatoxin biosynthesis, and fungal virulence in *Aspergillus flavus*

**DOI:** 10.3389/fcimb.2024.1372779

**Published:** 2024-03-26

**Authors:** Yarong Zhao, Chulan Huang, Rui Zeng, Peirong Chen, Kaihang Xu, Xiaomei Huang, Xu Wang

**Affiliations:** ^1^ Institute of Quality Standard and Monitoring Technology for Agro-product of Guangdong Academy of Agricultural Sciences, Guangzhou, China; ^2^ Guangdong Provincial Key Laboratory of Quality & Safety Risk Assessment for Agro-Products, Guangzhou, China; ^3^ Key Laboratory of Testing and Evaluation for Agro-product Safety and Quality, Ministry of Agriculture and Rural Affairs, Guangzhou, China

**Keywords:** *Aspergillus flavus*, aflatoxin biosynthesis, branched-chain amino acids, AflaILVB/G/I, AflaILVD, fungal secondary metabolites

## Abstract

Aflatoxins (AFs) are produced by fungi such as *Aspergillus flavus* and *A. parasiticus* and are one of the most toxic mycotoxins found in agricultural products and food. Aflatoxin contamination, which requires the control of *A. flavus*, remains problematic because of the lack of effective strategies and the exploration of new compounds that can inhibit *A. flavus* growth and mycotoxin production is urgently required to alleviate potential deleterious effects. Acetohydroxy acid synthase (AHAS) and dihydroxy acid dehydratase are important enzymes in the biosynthetic pathways of branched-chain amino acids (BCAAs), including isoleucine, leucine, and valine. Enzymes involved in BCAA biosynthesis are present in bacteria, plants, and fungi, but not in mammals, and are therefore, attractive targets for antimicrobial and herbicide development. In this study, we characterized *AflaILVB/G/I* and *AflaILVD*, which encode the catalytic and regulatory subunits of AHAS and dihydroxy acid dehydratase, from the pathogenic fungus *Aspergillus flavus*. The *AflaILVB/G/I* and *AflaILVD* deletion mutant grew slower and produced smaller colonies than the wild-type strain when grown on glucose minimal medium, potato dextrose agar, and yeast extract medium for three days at 28°C, and disruption of *AflaILVB/G/I* caused a significant reduction in conidia production when grown on all kinds of media. Cellular stress assays determined that all strains were sensitive to H_2_O_2_. Importantly, the pathogenicity and aflatoxin production were affected when *AflaILVB/G/I* and *AflaILVD* were knocked out, particularly *AflaILVB/G/I*. A series of genes that encoded enzymes involved in aflatoxin synthesis were downregulated, meaning that the knockout of *AflaILVB/G/I* influenced aflatoxin synthesis in *A. flavus* strain WT. Collectively, our results demonstrate the potential value of antifungals targeting *AflaILVB/G/I* in *A. flavus*.

## 1 Introduction

Mycotoxins are a wide variety of toxic secondary metabolites produced by fungi. Most are stable and heat-resistant, and are found in crops, feed, food, and other plant-derived products. Aflatoxins (AFs) are produced by fungi such as *Aspergillus flavus* and *A. parasiticus*. AFB_1_ is one of the most toxic mycotoxins and is classified as a 1A carcinogen by the International Center for Research on Cancer (Abdel-Wareth, 2023). It is the most common, serious, and widespread mycotoxin found in agricultural products and foods. Dietary exposure to AFs is estimated to affect more than five billion people globally (Strosnider et al., 2006). The most significant impact of AFs on human health is hepatocellular carcinoma (HCC). According to the World Health Organization (WHO), the number of deaths caused by HCC reached 830,000 globally in 2020, and AF is one of the most common risk factors for HCC in developing countries (WHO, 2022). AF pollution not only poses a serious threat to food safety but is also one of the factors affecting trade in various countries. In 2019, the EU Rapid Warning System for food and agricultural products from China to Europe recorded up to 30% of notifications associated with AFs. Therefore, research on control measures for AF pollution is of great significance for the food industry and consumer health protection.

Currently, the control of the AF pollution risk mainly focuses on detoxification technologies related to toxin pollution and the prevention and control of toxigenic fungi (Yu et al., 2020; Guan et al., 2021). Detoxification technology usually relies on physical, chemical, and biological methods to degrade toxins; however, such methods require the safety of the degradation products to be evaluated (Yang et al., 2022). Methods for controlling the growth of toxic fungi in edible agricultural products include the use of disease-resistant varieties, chemical agents, storage temperature and humidity controls, plant-derived bacteriostatic agents, and biological control (Xing et al., 2021). Although the development of antifungal agents has always been a focus of research, only a few agents are currently available. Therefore, the development of new agents is important for the continuous control of AF-producing fungi and AF pollution. AF contamination, which requires the control of *A. flavus*, remains problematic because of the lack of effective strategies. Thus, the exploration of new compounds that can inhibit *A. flavus* growth and mycotoxin production is urgently required to alleviate potential deleterious effects.

Amino acids are the basic building blocks of proteins that play an important role in the structure of body tissues and are essential for nutrition, metabolic regulation, immunity, and information transmission. Branched-chain amino acids (BCAAs) are synthesized in plants, fungi, bacteria, and algae but not animals. Therefore, a key enzyme in this synthetic pathway is a potential target for the development of non-toxic antimicrobial agents with limited toxicity in mammals.

Over the past 20 years, many drugs that have been developed and marketed worldwide, including sulfonylureas, imidazolinones, pyrimidine benzoates, triazolinones, sulfonamide-carbonyl triazolinones target acetohydroxy acid synthase (AHAS) in the BCAA pathway (Jeschke et al., 2019). Several studies have shown that some herbicide-targeting enzymes related to the BCAA pathway also exhibit antibacterial activity against human pathogenic bacteria. Lee et al. showed that chlorsulfuron (CE) had antibacterial effects on five different types of *Candida* and *Cryptococcus neoformans*. Garcia et al. further confirmed that the synergistic effect of CE and itraconazole effectively improves the survival rate of mice fed *Candida albicans* (Lee et al., 2013; Garcia et al., 2018). In addition, sulfonylureas exhibit antibacterial activity against *Mycobacterium tuberculosis*, which causes tuberculosis. For example, chloroxanthin has a stronger antibacterial effect against *M. tuberculosis* than streptomycin (Rehberg et al., 2018). In addition, studies have found that the BCAA synthesis pathway is an important part of mycotoxin biosynthesis, and the deletion of genes involved in the BCAA pathway causes mycotrophic deficiency, resulting in a significant reduction in pathogenicity and toxin biosynthesis (Du et al., 2013; Liu et al., 2019; Passalacqua et al., 2020).

In this study, we characterized the functions of *AflaILVB/G/I* and *AflaILVD*, which encode the catalytic and regulatory subunits of AHAS and dihydroxy acid dehydratase (DHD), respectively, using a strategy of targeted gene deletion and complementation in *A. flavus* to investigate their possible roles in various cellular processes, as well as their potential to serve as novel fungicide targets. Furtherly, transcriptome sequencing analysis of △*AflaILVB/G/I* was performed using the wild strain as a control group to investigate the regulatory mechanism.

## 2 Materials and methods

### 2.1 Strains, growth conditions, and phenotype assays

TJW 149.27 (*pyrG1, Δku70::AfpyrG*) was used as the parental wild-type (WT) strain. To assess mycelial growth and colony characteristics, the wild-type strain and mutants were cultured on different media, including glucose minimal medium (GMM), potato dextrose agar (PDA), yeast extract (YES), and yeast glucose trace (YGT), and incubated at 28°C. Colony diameter was measured at 28°C for three days. For the conidiation assay, three 5-mm mycelial plugs of the WT strain and mutants obtained from the edge of a three-day-old colony on PDA and YGT were placed in an Eppendorf tube containing 1 mL of normal saline, and the conidia were counted using a hemocytometer. The experiment was performed in triplicates.

### 2.2 Cellular sensitivity to different environmental stresses

To evaluate the sensitivity to various cellular stresses, 5-mm (diameter) mycelial plugs of the WT strain and mutants obtained from the edge of a three-day-old colony on PDA were transferred onto GMM with 8 mmol H_2_O_2,_ GMM + 1 M NaCl, GMM +1 M KCl GMM + 0.6% Congo red, and GMM + 0.3% calcofluor white (CFW). Colony morphology was photographed and the diameter was measured after incubation at 28°C for three days. The relative inhibition rates were calculated.

### 2.3 Targeted gene deletion

*AflaILVB/G/I* and *AflaILVD* deletion mutants were obtained as follows: The upstream and downstream homologous arm fragments of *AflaILVB/G/I* and *AflaILVD* were connected to the arg B fragment of *A. flavus* to construct knockout fragments through overlapping PCR. The knockout fragments were imported into the protoplasts of the TJES 20.1(Δku70/ΔargB) strain (kindly provided by Professor Yang, Jiangsu Normal University) through homologous recombination. Transformants positive for the knockout were identified through PCR and Southern blotting using arginine as a screening marker.

### 2.4 Peanut infection

The ability of WT and mutant strains to infect peanuts was assessed. WT and mutant strain conidia were harvested from three-day-old PDA cultures, washed with sterilized water, and resuspended in 0.01% (v/v) Tween 20 solution. The concentration of the conidial suspension was adjusted to 1 × 10^7^ conidia/mL. Peanut seeds weighing approximately 20 g were sterilized with 75% medical alcohol for 1 min and then rinsed five times with sterile water so that the water content of the seeds reached approximately 20%. Each peanut was soaked in spore suspension for 5 s, placed in a disposable petri dish, sealed, and placed in an incubator at 28°C for five days. The experiment was repeated three times.

### 2.5 AFB1 analysis

To determine AFB1 production, 10-mL aliquots of 1 × 10^6^ spore/mL suspension of *A. flavus* conidia of the WT and mutant strains were incubated in YES and PDB media in the dark at 28°C for 3.5 d. Thin layer chromatography (TLC) was used to analyze AF production as previously described (Yang et al., 2016). AF was extracted from the 500-μL filtrate with an equal volume of chloroform. The chloroform layer was transferred to a new 1.5 mL tube, then evaporated to dryness at 70°C. Next, TLC was used to analyze AF biosynthesis. A solvent system consisting of acetone and chloroform (1:9, v/v) was used, and TLC results were obtained under ultraviolet light at 365 nm.

For the quantitative analysis of AF production, high-performance liquid chromatography-tandem mass spectrometry (HPLC-MS/MS) was used to confirm the presence of AF in infected peanuts. The peanuts were sterilized at 121°C for 30 min before grinding, and the analysis performed as previously described (Zhao et al., 2017).

### 2.6 cDNA preparation and Illumina sequencing

Total RNA was extracted using a Total RNA Extractor (TRIzol) kit (B511311, Sangon Biotech Co. Ltd., Shanghai, China) according to the manufacturer´s protocol and treated with RNase-free DNase I to remove genomic DNA contamination. RNA integrity was evaluated using a 1.0% agarose gel electrophoresis. The quality and quantity of RNA were assessed using a NanoPhotometer ^®^ spectrophotometer (Implen, Westlake Village, CA, USA) and a Qubit^®^ 2.0 Fluorometer (Invitrogen). High-quality RNA samples were subsequently submitted to Sangon Biotech Co. Ltd., China) for library preparation and sequencing.

Briefly, mRNA was purified from the total RNA using poly-T oligo-attached magnetic beads. Sequencing libraries were generated using VAHTSTM mRNA-seq V2 Library Prep kit for Illumina^®^ following manufacturer´s recommendations and index codes were added to attribute sequences to each sample. Fragmentation was conducted using divalent cations at elevated temperatures in VAHTSTM First Strand Synthesis Reaction Buffer (5X). First strand cDNA was synthesized using random hexamer primer and M-MuLV reverse transcriptase (RNase H-). Second-strand cDNA synthesis was subsequently performed using DNA polymerase I and RNase H. Remaining overhangs were converted into blunt ends via exonuclease/polymerase activity. After adenylation of 3´ ends of DNA fragments, an adaptor was ligated to prepare the library. To select cDNA fragments preferentially 150–200 bp in length, the library fragments were purified using the AMPure XP system (Beckman Coulter, Brea, C A, USA). Then, 3 μL USER Enzyme (NEB, USA) was used with size-selected, adaptor-ligated cDNA at 37°C for 15 min followed by 5 min at 95°C before PCR. PCR was performed using the Phusion High-Fidelity DNA polymerase, universal PCR primers, and Index (X) Primer. Finally, the PCR products were purified (AMPure XP system), and library quality was assessed using an Agilent Bioanalyzer 2100 system. The libraries were quantified and pooled. Paired-end sequencing of the library was performed on a NovaSeq sequencer (Illumina, San Diego, CA, USA).

### 2.7 Expression analysis

The gene expression of the transcripts was calculated using StringTie (version 1.3.3b). A principal component analysis (PCA) was performed to determine the distance between samples. Transcripts per million (TPM) eliminated the influence of gene length and sequencing discrepancies to enable a direct comparison of gene expression between samples. DESeq2 (version 1.12.4) was used to determine differentially expressed genes (DEGs) between two samples. Genes were considered as significantly differentially expressed if the q-value was < 0.05 and |FoldChange| > 2.

### 2.8 Functional analysis of DEGs

Functional enrichment analyses, including Gene Ontology (GO) and the Kyoto Encyclopedia of Genes and Genomes (KEGG), were performed to identify which DEGs were significantly enriched in GO terms or metabolic pathways. GO is an international standard classification system for gene functions. DEGs were mapped to GO terms (biological functions) in the database, the number of genes in each term was calculated, and a hypergeometric test was performed to identify significantly enriched GO terms in the gene list from the background of the reference gene list. The KEGG database is a public database of pathway data, and KEGG pathway analysis identifies significantly enriched metabolic or signal transduction pathways enriched in DEGs compared to a reference gene background using the hypergeometric test. GO terms and KEGG pathways with a false discovery rate (q-value) < 0.05 were considered significantly altered.

### 2.9 Analysis of gene expression

Total RNA of the WT and △*AflaILVB/G/I* was extracted and cDNA was reverse transcribed using fungal RNA extraction kit (Coolaber, Beijing, China) and Hifair^®^ III 1st Strand cDNA Synthesis kit (Yeasen Biotech Co. Ltd., Shanghai, China). Expression of the genes involved in aflatoxins synthesis was determined by quantitative real-time PCR (RT-PCR) with primes (**Table 1**). The RT-PCR amplifications were performed in a QuantStudio 3 (Thermo Fisher, Massachusetts, USA) using SYBR Green I fluorescent dye detection. Amplifications were conducted in a 20 ml volume containing 10 ml iQ SYBR Green Supermix (Bio-Rad Laboratories), 2 ml reverse transcription product and 0.4 ml of each primer. There were three replicates for each sample. RT-PCR amplifications were performed with the following parameters: an initial preheat at 95°C for 2 min, followed by 40 cycles at 95°C for 10 s, 60°C for 30 s. Once amplifications were completed, melting curves were obtained to identify PCR products. For each sample, PCR amplifications with primer pair actin of *A.flavus* for quantifying expression of the actin gene were performed as a reference. The experiment was repeated three times. Levels of expression of the genes of interest in the WT strain and deletion mutants were calculated using the 2-ΔΔCT method (Livak and Schmittgen, 2001).

**Table 1:** Prime sequence of gene for real-time PCR.

Gene	Prime sequence(5’-3’)
actin/QF	ACGGTGTCGTCACAAACTGG
actin/QR	CGGTTGGACTTAGGGTTGATAG
aflQ/QF	GTCGCATATGCCCCGGTCGG
aflQ/QR	GGCAACCAGTCGGGTTCCGG
aflK/QF	CTCGCACTTTGGCATGTACG
aflK/QR	AATCCTCCCGCCTCAATCAC
alfH/QF	CCAATCCGTCCCCTGACAAG
alfH/QR	CCGAGTGCTGCGAAATAACC
hypC/QF	GCATGGTGCCTTACACATGG
hypC/QR	CCTACCAACCTCACGCTCTC
aflD/QF	GTGGTGGTTGCCAATGCG
aflD/QR	CTGAAACAGTAGGACGGGAGC

## 3 Results

### 3.1 Identification and analysis of *AflaILVB/G/I* and *AflaILVD* in *A. flavus*

Amino acids are important components in the metabolism of a variety of pathogens, plants, and animals. *AFLA_000930* and *AFLA_003530*, orthologs of *S. cerevisiae ILV2* and *ILV3* and involved in valine, leucine, and isoleucine biosynthesis, were identified through BLAST analysis and named *AflaILVB/G/I* and *AflaILVD*. The regulatory subunit of AHAS AflaILVB/G/I is one of several enzymes that require thiamine pyrophosphate (TPP, vitamin B_1_) as a cofactor. Some of these enzymes were structurally related (PubMed: 8604141). All of them have an N-terminal central domain, and C-terminal TPP-binding domain for TPP enzymes. *AflaILVD* encodes a dihydroxy-acid dehydratase and catalyzes the fourth step in the biosynthesis of isoleucine and valine, that is, the dehydration of 2,3-dihydroxy-isovaleic acid into alpha-ketoisovaleric acid. The enzyme 6-phosphogluconate dehydratase catalyzes the first step in the Entner-Doudoroff pathway, that is, the dehydration of 6-phospho-D-gluconate into 6-phospho-2-dehydro-3-deoxy-D-gluconate. Another protein with this signature is *Escherichia coli* YjhG, which has been identified as a d-xylonate dehydratase. The N-terminal region of the protein contains a cysteine residue that may be involved in the binding of a 2Fe-2S iron-sulfur cluster.

### 3.2 *AflaILVB/G/I* and *AflaILVD* are involved in colony growth and conidiation in *A. flavus*

**Figure 1:**
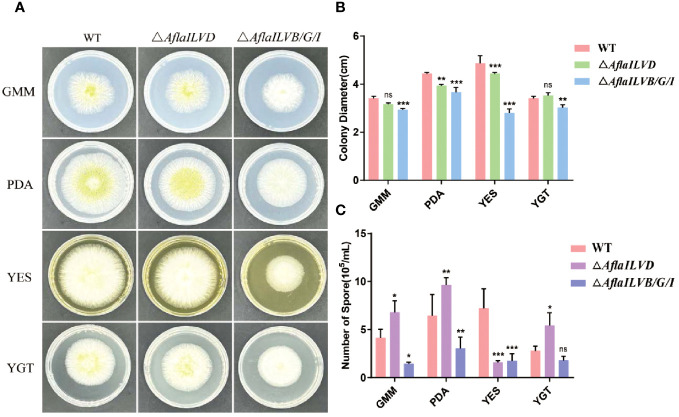
Influence of *AflaILVB/G/I* and *AflaILVD* on colony growth and conidiation. **(A)** Colonies formed by the WT, △*AflaILVB/G/I*, and △*AflaILVD* strains on GMM, PDA, YES, and YGT medium agar plates. **(B)** Colony diameters of WT, △*AflaILVB/G/I*, and △*AflaILVD* strains on GMM, PDA, YES, and YGT medium agar plates. **(C)** Number of spores of WT, △*AflaILVB/G/I*, and △*AflaILVD* strains on GMM, PDA, YES, and YGT media. *: p<0.05, **: p<0.01, ***: p<0.001.

To gain insight into the possible functions of *AflaILVB/G/I* and *AflaILVD* in the developmental stages of *A. flavus*, we generated △*AflaILVB/G/I* and △*AflaILVD* in the WT strain. As shown in **Figures 1A, B**, when grown on GMM, PDA, and YES medium for 3 days at 28°C, the △*AflaILVB/G/I* and △*AflaILVD* mutants grew slower and produced smaller colonies than the WT strain. When grown on YGT medium, however, the colony diameter of the △*AflaILVD* mutant was greater than that of the WT. In addition, △*AflaILVB/G/I* was significantly different compared with △*AflaILVD.*

**Figure 1C** shows that the disruption of *AflaILVB/G/I* caused a significant reduction in conidial production when grown on all media, but it is interesting that when grown on GMM, PDA, and YGT media, the △*AflaILVD* mutant produced more conidia than the WT strain, but it produced fewer on the YES medium.

### 3.3 *AflaILVB/G/I* and *AflaILVD* are required for adaptation to various cellular stresses in *A. flavus*

**Figure 2:**
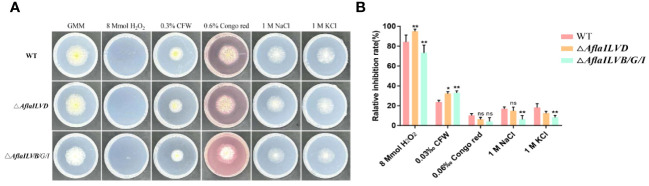
**(A)** Phenotype and **(B)** relative inhibition rate (%) of WT, △*AflaILVB/G/I*, and △*AflaILVD A. flavus* strains grown on GMM medium under various cellular stresses. *: p<0.05, **: p<0.01, ns: no significance.

To test the responses of *AflaILVB/G/I* and *AflaILVD* to various environmental stresses, the sensitivities of the WT and mutant strains to osmotic and oxidative stresses were assessed. As shown in **Figures 2A** and **2B**, when compared with WT, △*AflaILVB/G/I* and △*AflaILVD* strains did not exhibit recognizable changes to cell-wall-damaging agents Congo red (0.6%), 1 M NaCl and GMM + 1 M KCl. However, all strains were sensitive to 8 mmol H_2_O_2_ and 0.3% CFW. For H_2_O_2_, the relative inhibition rate of *△AflaILVB/G/I* was > 70%.

### 3.4 *AflaILVB/G/I* and *AflaILVD* participate in full virulence and AFB1 biosynthesis in *A. flavus*

**Figure 3:**
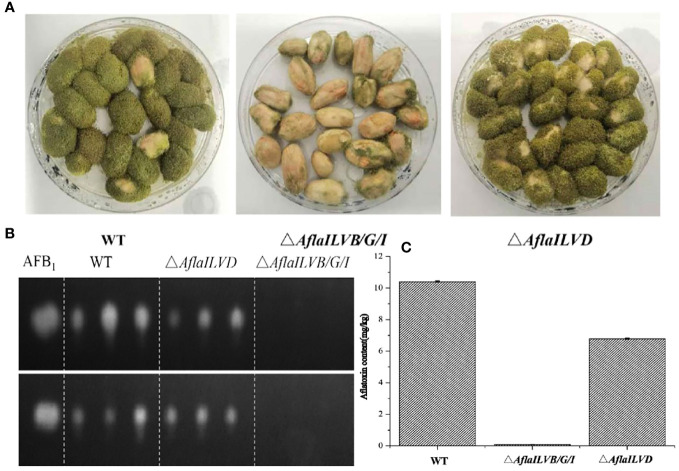
**(A)** Pathogenicity for peanut seed and **(B)** AFB1 production by WT, △*AflaILVB/G/I*, and △*AflaILVD* strains used TCL, **(C)** AF production by WT, △*AflaILVB/G/I*, and △*AflaILVD* strains used HPLC-MS/MS.

To explore the function of *AflaILVB/G/I* and *AflaILVD* genes in the pathogenicity and AF biosynthesis in *A. flavus*, we inoculated peanuts with WT and mutant strains. Peanut infection assays revealed the crucial role of *AflaILVB/G/I* and *AflaILVD* in full virulence and mycotoxin biosynthesis. As shown in **Figure 3A**, the pathogenicity of the mutants lacking *AflaILVB/G/I* and *AflaILVD* was reduced, and the former exhibited a greater loss of virulence. **Figures 3B, C** show that AFB1 production was detected by TLC and HPLC-MS/MS in the WT, △*AflaILVB/G/I*, and △*AflaILVD* strains. The synthesized AFB1 content was affected when *AflaILVB/G/I* and *AflaILVD* were knocked out, especially for *AflaILVB/G/I*, for which AFB1 production was significantly different from that of the WT.

### 3.5 Quality control and gene expression analysis

**Figure 4:**
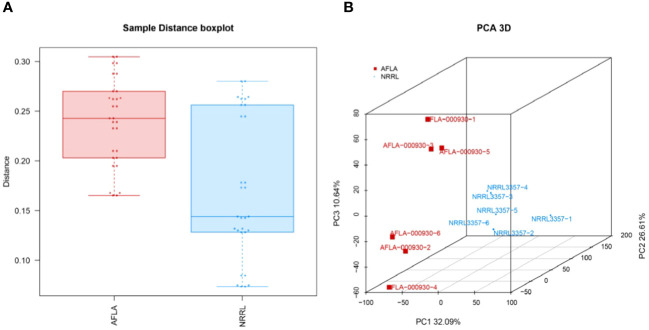
**(A)** TPM distribution boxplot and **(B)** PCA analysis. Points of different colors or shapes represent different groups, and the scale of the horizontal and vertical coordinate axes represents the relative distance, which has no practical significance.

The statistical findings of the sequencing data for the clean reads of the final obtained were Q20 > 95%. Q30 > 89%, indicating that the sequencing quality was good and that it could be used for subsequent comparative and data analyses. The results of the gene expression analysis and PCA are shown in **Figure 4**. The close distance between the samples within the groups indicated a high similarity between the samples, and the distance between the samples indicated that there were significant differences between the groups.

### 3.6 DEGs identification and analysis

**Figure 5:**
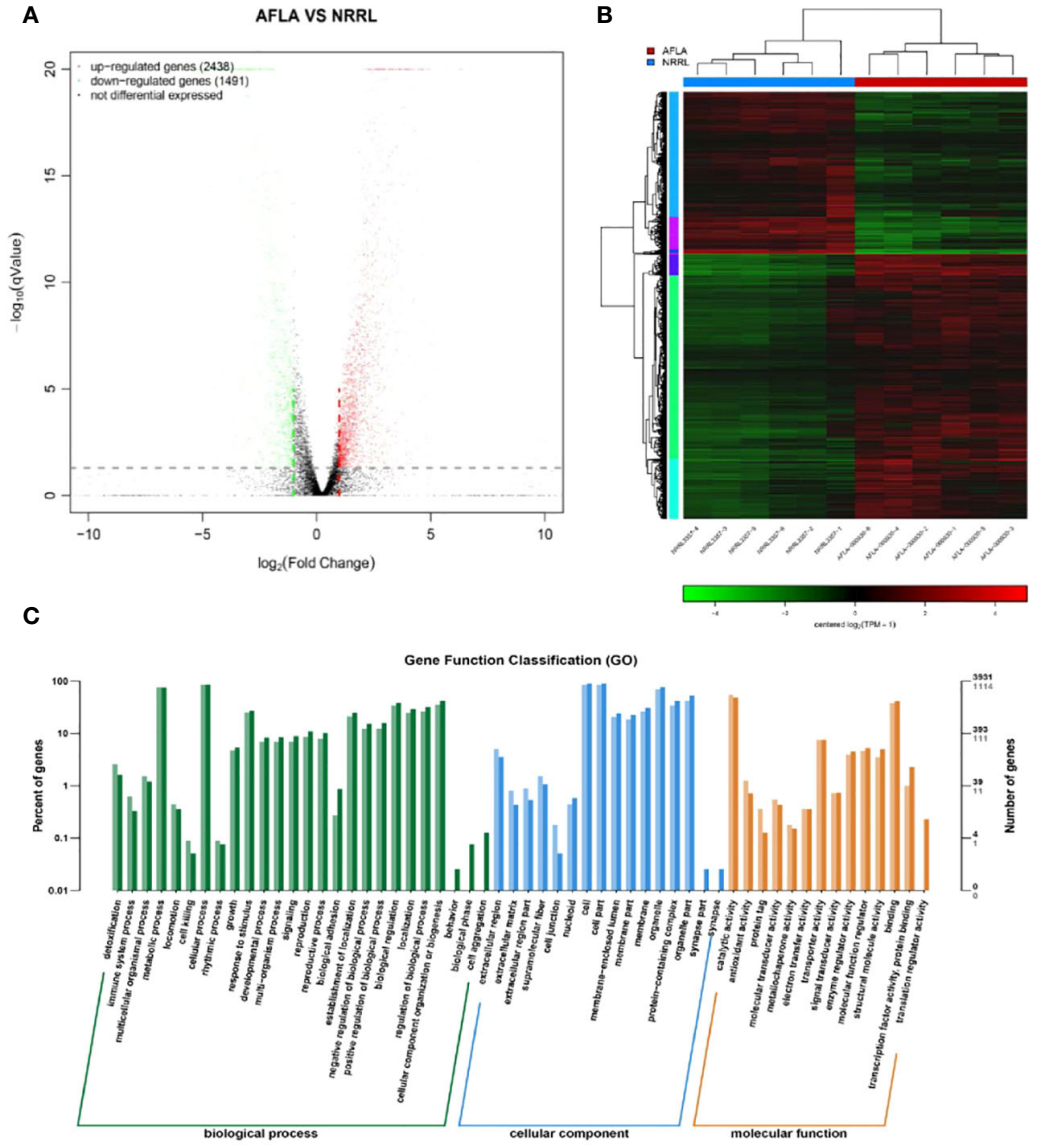
**(A)** Volcano map of the differentially expressed genes **(B)** Clustering results of AFLA and NRRL **(C)** GO functional enrichment analysis of differentially expressed genes.

To identify the differences in molecular responses between the WT and mutant strains, TMM was used to standardize the read count and DEGseq was used to analyze the differences (q-value < 0.05, |FoldChange| > 2); 3929 DEGs were obtained. Among them, 2438 and 1491 genes were upregulated and downregulated, respectively (**Figure 5A**).

The expression levels of differential genes under different experimental conditions were used for hierarchical clustering analysis. The biological function of a gene was predicted by grouping genes with high expression correlations in different groups. Genes with similar colors have similar expression patterns in the cluster area; the similarity of these patterns suggests that these genes may have similar functions or be involved in regulating the same metabolic pathway (**Figure 5B**).

Functional assignments were defined using GO terms, which provided a comprehensive functional classification of genes and their products based on various biological processes, cellular components, and molecular functions. GO functional enrichment analysis revealed several enriched terms for DEGs. **Table 2** shows the top 10 most significantly different GO terms. Among them, small-molecule metabolic, carboxylic acid metabolic, oxoacid metabolic, organic acid metabolic, and small-molecule catabolic processes were the most significant. In addition, as shown in **Figure 5C**, according to the number of genes enriched in GO terms, most DEGs were enriched in cellular processes of biological processes, followed by cell and cell parts of cellular components.

**Table 2:** GO functional enrichment of top 10 most significant differentially expressed genes.

GO.ID	Term	Ontology	Qvalue
GO:0044281	small molecule metabolic process	biological process	1.71E-09
GO:0019752	carboxylic acid metabolic process	biological process	4.79E-08
GO:0043436	oxoacid metabolic process	biological process	2.40E-07
GO:0006082	organic acid metabolic process	biological process	2.40E-07
GO:0044282	small molecule catabolic process	biological process	4.93E-05
GO:0044438	microbody part	cellular component	0.000102085
GO:0044439	peroxisomal part	cellular component	0.000102085
GO:0006631	fatty acid metabolic process	biological process	0.000125547
GO:0032787	monocarboxylic acid metabolic process	biological process	0.000391314
GO:0005777	peroxisome	cellular component	0.000870725

Pathway-based analysis was performed using the KEGG pathway database to explore the biological functions and interactions in the genes. The results showed that 3929 DEGs were involved in 261 pathways. **Table 3** shows the top ten upregulated and downregulated genes enriched in KEGG. Among the downregulated genes, the most enriched pathways were those involved in AF biosynthesis, followed by serotonergic synapses and pyruvate metabolism. This suggests that the expression of genes involved in AF synthesis was suppressed in the experimental group. In contrast, the most enriched pathway among the upregulated genes was peroxisomes, followed by purine and sulfur metabolisms. This indicates that genes encoding oxidation-reduction enzymes were highly expressed in the experimental groups. Furthermore, KEGG metabolic pathway analysis confirmed that the enrichment of peroxisomes, sulfur metabolism, and AF biosynthesis was significant *(p <* 0.05).

**Table 3:** Top 10 KEGG enrichment for down and upregulated genes.

id	Description	P-value
Downregulated genes		
ko00254	Aflatoxin biosynthesis	2.46E-09
ko04726	Serotonergic synapse	0.006026125
ko00620	Pyruvate metabolism	0.006148805
ko00361	Chlorocyclohexane and chlorobenzene degradation	0.006152135
ko04211	Longevity regulating pathway - mammal	0.006340702
ko04212	Longevity regulating pathway - worm	0.00959147
ko00500	Starch and sucrose metabolism	0.011060419
ko00710	Carbon fixation in photosynthetic organisms	0.013884751
ko00791	Atrazine degradation	0.01530548
ko04913	Ovarian steroidogenesis	0.01530548
Upregulated genes		
ko04146	Peroxisome	1.08E-06
ko00230	Purine metabolism	9.14E-05
ko00920	Sulfur metabolism	0.000388066
ko00670	One carbon pool by folate	0.00051374
ko00280	Valine, leucine and isoleucine degradation	0.000822652
ko04122	Sulfur relay system	0.000830843
ko00071	Fatty acid degradation	0.002479551
ko00970	Aminoacyl-tRNA biosynthesis	0.002525035
ko00240	Pyrimidine metabolism	0.003436001
ko00270	Cysteine and methionine metabolism	0.0034424

### 3.7 Analysis of DEGs involved in AF biosynthesis

**Figure 6:**
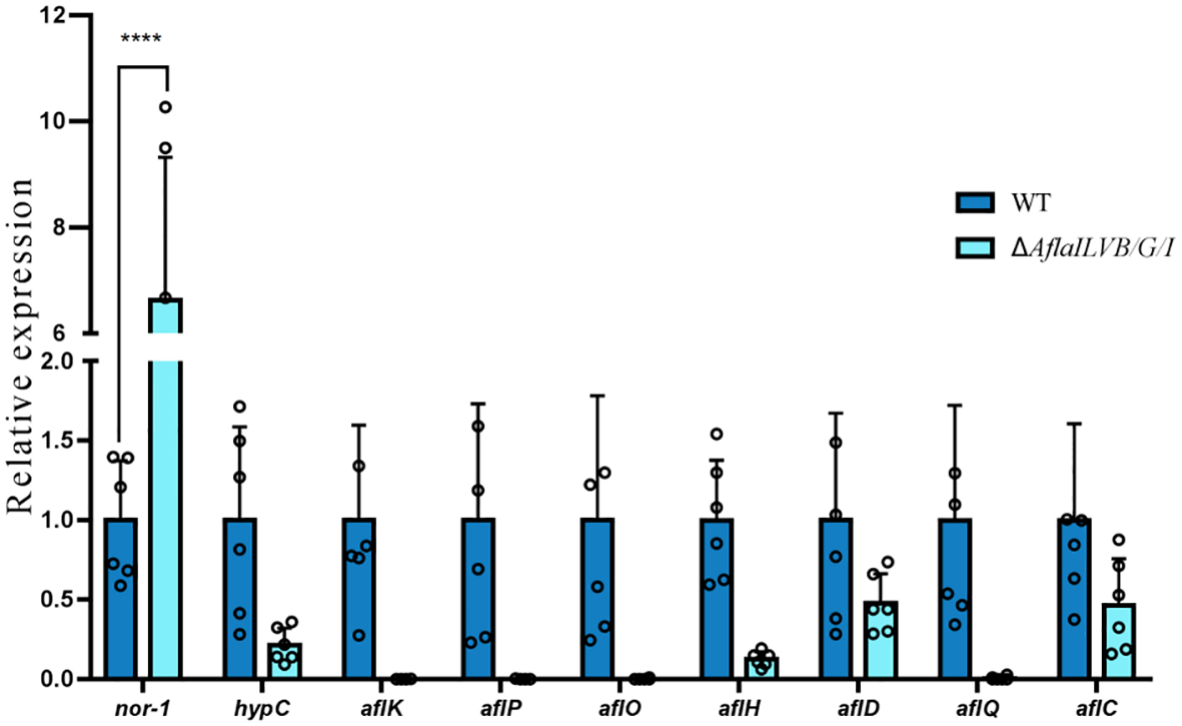
Differentially expressed genes involved in aflatoxin biosynthesis. ****: p<0.0001.

The DEGs involved in aflatoxin biosynthesis are shown in **Figure 6**, which shows that among the ten differentially expressed genes annotated in the two strains, eight genes were downregulated: hypC, *aflK*, *aflP, aflO*, *aflH*, *aflD*, *aflC*, *aflQ* and *nor-1* was also upregulated, and one gene remained unchanged.

**Figure 7:**
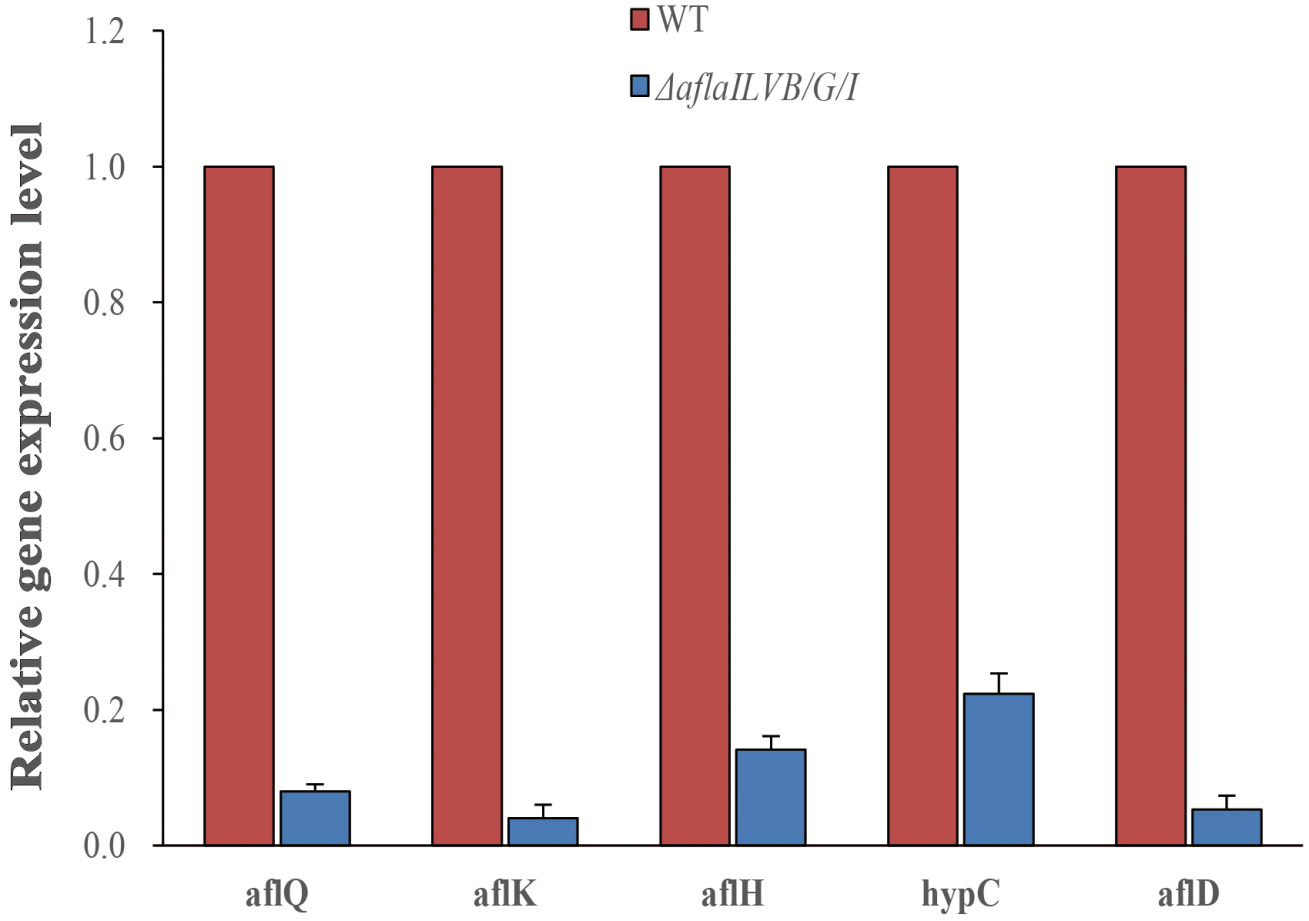
Relative expression of AFB_1_ synthetic genes in the △*AflaILVB/G/I*.

To further confirm the results, we assayed the expression of aflQ, aflK, aflH, hypC, aflD by quantitative RT-PCR using RNA samples isolated from mycelia grown in PDB medium for 4 days at 28°C. The expression level of aflQ, aflK, aflH, hypC and aflD in the △*AflaILVB/G/I* was decreased by 92, 96, 86, 78 and 95%, respectively, as compared with levels in the WT (**Figure 7**).

## 4 Discussion

AHAS is the first shared enzyme in the BCAA biosynthetic pathway and is an inhibitory target of the most widely used herbicides (AHAS inhibitors) (Lonhienne et al., 2018). Some AHAS-targeted herbicides exert inhibitory effects against several pathogenic bacteria and yeast, as reported in previous studies (Kreisberg et al., 2013), indicating a promising potential for AHAS as an anti-microorganism target. Functional analyses of *S. cerevisiae*, *C. albicans*, *C. neoformans*, *M. oryzae*, and *F. graminearum* have suggested that the AHAS catalytic subunit-encoding gene ILV2 has the potential to be an antifungal target, based on phenotypic defects caused by the disruption of ILV2 (Kingsbury et al., 2004, 2006; Kingsbury and McCusker, 2010; Liu et al., 2015). In this study, the blocking of BCAA biosynthesis caused by the deletion of *AflaILVB/G/I* and *AflaILVD* resulted in defects in vegetative growth and conidiation. Based on the degree of severity of phenotypic defects displayed by the mutants, *AflaILVB/G/I* may play a more important role in *A. flavus* than *AflaILVD*. In addition, pathogenicity and AFB_1_ accumulation were impaired in both deletion mutants. In the infection assay on peanut, the virulence of △*AflaILVB/G/I* and △*AflaILVD* strains was reduced and the former was almost completely lost.

*In vitro*, the deletion mutants were associated with severely infected phenotypes; therefore, it was not possible to assess their relative virulence. Compared with WT strains, △*AflaILVB/G/I* colony growth was reduced and fewer spores were observed when cultured on a medium. In addition, reactive oxygen species play essential roles in host-pathogen interactions. During a pathogen attack, organisms use an oxidative burst as an early defense mechanism (Liu et al., 2014). △*AflaILVB/G/I* showed significantly increased sensitivity to the oxidative agent H_2_O_2_, which may be related to the reduced virulence in peanut. The deletion mutants were less sensitive than the WT to osmotic stress mediated by Congo red, NaCl, and KCl.

Based on phenotypic analysis, △*AflaILVB/G/I* and WT strains were transcriptome sequenced, a total of 3929 differential genes were screened, of which 2438 upregulated and 1491 downregulated genes. GO and KEGG enrichment analyses were performed on DEGs. More importantly, in our study, a series of genes that encode enzymes involved in aflatoxin synthesis were downregulated, indicating that *AflaILVB/G/I* knockout influenced aflatoxin synthesis in *A. flavus* strain NRRL3557.

AF contamination remains a major global food safety challenge with serious implications for agriculture and public health (Nzuma and Mzera, 2023). *A. flavus* is a representative fungal species in *Aspergillus* section Flavi and has been used as a model system to gain insights into fungal development and toxin production. In this study, by identifying the key genes in the BCAA synthesis pathway of *A. flavus* and analyzing their biological functions, we clarified the roles of BCAAs in the important physiological processes of growth, sporulation, pathogenicity, and AF synthesis. This study provides a theoretical basis for developing new antimicrobials to reduce AF pollution.

In conclusion, *ILV* may be a key gene affecting the growth, development, pathogenicity, and toxin synthesis in *A. flavus*. The key genes in this pathway can be used to develop new, safe antimicrobial agents to prevent and control AF contamination.

## Data availability statement

The data presented in the study are deposited in the Genome Sequence Archive (Genomics, Proteomics & Bioinformatics 2021) in National Genomics Data Center repository, accession number CRA015215.

## Author contributions

YZ: Conceptualization, Writing – original draft. CH: Methodology, Writing – review & editing. RZ: Formal analysis, Writing – review & editing. PC: Methodology, Writing – review & editing. KX: Validation, Writing – review & editing. XH: Validation, Writing – review & editing. XW: Writing – review & editing.
